# Pediatric ADHD symptom burden relates to distinct neural activity across executive function domains

**DOI:** 10.1016/j.nicl.2020.102394

**Published:** 2020-08-25

**Authors:** Tehila Nugiel, Mary Abbe Roe, Laura E. Engelhardt, Mackenzie E. Mitchell, Annie Zheng, Jessica A. Church

**Affiliations:** a Department of Psychology, The University of Texas at Austin, Austin, TX, United States; b Department of Psychology and Neuroscience, University of North Carolina at Chapel Hill, Chapel Hill, NC, United States; c Department of Neurology, Washington University School of Medicine, St. Louis, MO, United States; d Biomedical Imaging Center, The University of Texas at Austin, Austin, TX, United States

**Keywords:** ADHD, fMRI, Development, Executive functions, Hyperactivity, Inattention

## Abstract

Attention-deficit/hyperactivity disorder (ADHD) is a prevalent childhood disorder marked by inattention and/or hyperactivity symptoms. ADHD may also relate to impaired executive function (EF), but is often studied in a single EF task per sample. The current study addresses the question of unique *vs*. overlapping relations in brain activity across multiple EF tasks and ADHD symptom burden. Three in-scanner tasks drawn from distinct EF domains (cognitive flexibility, working memory, and inhibition) were collected from children with and without an ADHD diagnosis (N = 63). Whole-brain activity and 11 regions of interest were correlated with parent reports of inattention and hyperactivity symptoms. Across the three EF domains, brain activity related to ADHD symptom burden, but the direction and location of these associations differed across tasks. Overall, activity in sensory and default mode network regions related to ADHD, and these relations did not consistently overlap across EF domains. We observed both distinct and overlapping patterns for inattention and hyperactivity symptoms. By studying multiple EF tasks in the same sample, we identified a heterogenous neural profile related to attention symptom burden in children. Our results inform ADHD characterization and treatment and explain some of the variable brain results related to EF and ADHD reported in the literature.

## Highlights

• ADHD symptoms relate differently to brain activity across executive function tasks.
• Hyperactivity and inattention show unique and overlapping relations to brain activity.
• Activity in sensory and default mode network regions related to ADHD symptoms.
• Dimensional and categorical models of ADHD reveal different effects.

## 1 Introduction

Attention-deficit/hyperactivity disorder (ADHD) is a neurodevelopmental disorder diagnosed in an estimated 9% of the school-aged population in the US (Danielson et al., 2018). Pediatric ADHD is primarily characterized by inattention and hyperactivity/impulsivity symptoms (American Psychiatric Association, 2013, Cortese, 2012, Friedman and Rapoport, 2015, Willcutt et al., 2005b, Willcutt et al., 2005c). This symptom burden can impact cognitive, academic, and social-emotional processes (Chacko et al., 2014, Cortese, 2012, Nigg et al., 2005, Voeller, 2004). ADHD is highly comorbid with learning difficulties (Langer et al., 2019; Willcutt et al., 2010), and some individuals with ADHD have impairments in executive functions (EFs) (Biederman, 2003, Kofler et al., 2018, Nigg et al., 2005, Roberts et al., 2017). EFs support goal-oriented behaviors and comprise regulatory processes across several domains such as cognitive flexibility, working memory, and inhibition (Diamond, 2013, Miyake and Friedman, 2012) and are vital in the transition from immature to mature cognition and behaviors (Diamond, 2013, Miyake and Friedman, 2012). Behavioral models of EF support the idea that while an individual is consistent in their EF abilities, there may be variability across different components or domains, reflecting both unity and diversity (Miyake and Friedman, 2012). This variability could be particularly relevant to EF impairments in ADHD, which itself is phenotypically heterogenous, with estimates of EF impairments in ADHD ranging from 35 to 80% (Biederman et al., 2004, Happe et al., 2006, Kofler et al., 2018, Nigg et al., 2005, Roberts et al., 2017, Willcutt et al., 2005a).

The unity of EF is reflected in the organization of neural systems that support these processes across domains. Tasks that tap EF reliably recruit particular frontal and parietal brain regions across ages (Dosenbach et al., 2007, Engelhardt et al., 2019). These regions make up putative task control networks that cluster together during task and rest, such as the fronto-parietal network thought to support flexible shifts in attentional resources, and the cingulo-opercular network thought to support sustained attention (Power & Petersen, 2013). These networks are well established in adults (Petersen & Posner, 2012), and components of these networks, termed ‘core’ EF control regions, are reliably engaged by control-demanding tasks across EF domains by middle childhood (Engelhardt et al., 2019).

Given the existence of a consistent neural system supporting EFs in childhood, along with evidence of EF impairments in pediatric ADHD (Kofler et al., 2018, Nigg et al., 2005, Roberts et al., 2017), control-demanding tasks are ideal paradigms for studying variation in brain activity in children with ADHD symptom burden. This approach is common in the ADHD literature (Banich et al., 2009, Cortese et al., 2012, Depue et al., 2010, Fassbender et al., 2009, Peterson et al., 2009, Rubia et al., 2009a). However, findings vary from study to study, with many different brain regions and functional brain networks reported to be impacted in ADHD across the literature. Structure and function of task-control and attention networks are indeed often reported to vary in samples with ADHD (Cortese et al., 2012, Rubia et al., 2012, Rubia et al., 2014). Neural activity in the default mode network (DMN) is also reported to vary with ADHD; this network is characterized by positive/increased activity during rest or task-absent periods and suppressed (more negative) activity during task execution (Castellanos and Proal, 2012, Friedman and Rapoport, 2015, Raichle et al., 2001, Raichle and Snyder, 2007, Rubia et al., 2014: Sonuga-Barke and Castellanos, 2007). Finally, striatal and motor regions are also reported to show differing patterns of activity as a function of ADHD symptomatology (Castellanos and Proal, 2012, Friedman and Rapoport, 2015, Rubia et al., 2014).

There is immense breadth in the brain regions named in disrupted EFs among children with ADHD, but there is a surprising lack of depth; the same regions or networks are not consistently implicated across studies. Across the three-domain model of EF, inhibition tasks are most commonly used to look at brain function related to ADHD (Cortese, 2012, Rubia et al., 2003), with fewer studies employing working memory (Banich et al., 2009, Depue et al., 2010) and switching tasks (Cubillo et al., 2010). Importantly, most studies have used a single EF task representing a single domain to examine neural activity in their samples. Marked variability across study parameters and individual EF tasks generates an open question: Within a single sample, does ADHD symptomology relate to different facets of EF in a similar way? Behavioral EF heterogeneity in pediatric ADHD (Kofler et al., 2018, Roberts et al., 2017) suggests potential distinct neural patterns of ADHD across EF domains; however, support for this hypothesis would require testing multiple domains in a single sample.

The current study used a pediatric dataset to look for consistency of ADHD symptom impact across three MRI tasks sampled from separate EF domains. Neural activity from each task was related to parent reports of child inattention and hyperactivity, as measured on a continuous scale. Analyzing ADHD symptom burden as a continuous phenotype, rather than with the presence or absence of a diagnostic label, has proven to be a strong approach to capturing variability within the disorder and in the general population (Depue et al., 2010, Erway et al., 2019). We examined the question of consistent ADHD symptom burden impact on EF neural engagement across individuals with and without an ADHD diagnosis. We defined symptom burden as parent reports of behavioral dimensions of inattention and hyperactivity associated with ADHD, regardless of diagnostic status. By looking across three tasks within one sample, we controlled for many confounds that may contribute to the inconsistent findings of brain activity during EF tasks varying with ADHD in the literature. We used this approach to address two primary questions: 1) Do inattention and hyperactivity symptoms covary with EF brain function in the same way, i.e., do they impact the same EF domains? 2) Do ADHD symptoms impact the ‘core’ control regions engaged across multiple EF tasks, or do they impact task-specific regions?

## 2 Methods & Materials

### 2.1 Participants

Participants were drawn from a broad community collection assessing EF development in brain and behavior. The current sample (total N = 63, ages 8–18 years) was 68.3% white, 30.2% Hispanic (including Hispanic multiracial), 4% African American, and 1.5% Native American. All data collection procedures followed the human subjects research regulations overseen by the University of Texas at Austin Institutional Review Board. Parents provided informed consent for children less than age 18 years, and children under 18 provided informed assent. Families participated in one behavioral visit and one MRI visit. Participants were compensated for their time, and parents/guardians were compensated for completing research forms.

The sample was made up of typically developing children who had no diagnosed developmental or psychological disorders (N = 28, 35.71% female, mean age = 12.31 years *SD* = 2.53) and children with an ADHD diagnosis, including ADHD comorbid with other neuropsychological disorders (N = 35, 34.29% female, mean age = 12.65 years SD = 3.05). See Table 1 and Supplemental Tables 1 and 2 for group details. A subgroup of individuals with only an ADHD diagnosis and who were not medicated at scan time was used for more restrictive analyses (‘ADHD-only subgroup’, N = 17, 35.3% female, mean age = 12.65 years SD = 2.63). ADHD diagnosis status and date of diagnosis were reported by the parent.

**Table 1:** Participant group demographics and task performance.

	ADHD (N = 35)	Typical (N = 28)	ADHD restricted subgroup (N = 17)
Mean age in years (SD)	12.65 (3.05)	12.31 (2.53)	12.65 (2.63)
Age range	8.05–18.62	8.66–17.11	9.18–17.2
% female	34.29 (12)	35.71 (10)	35.3 (6)
Mean IQ	114.23	110.82	115.41
Mean CF accuracy	0.90	0.91	0.92
Mean CF RT correct trials	0.920	0.888	0.906
CF RT variability (SD)	0.301	0.304	0.288
Mean NB 2back accuracy	0.87	0.88	0.86
Mean NB 2back RT, correct hits	0.799	0.719	0.841
NB 2back RT variability (SD)	0.277	0.279	0.278
Mean SSRT	0.221	0.239	0.218
Mean SST Go accuracy	0.87	0.91	0.84
SST Go RT variability (SD)	0.127	0.126	0.125
Mean total raw Parent rated Conners-3	34.29 (11.08)***	14.5 (10.55)	35.47 (10.46)***

The ADHD restricted subgroup included individuals with no comorbid diagnoses and who were unmedicated during the scan; ADHD = attention deficit hyperactivity disorder; SD = standard deviation; RT = response time; IQ = age-normed score on the Weschler’s Abbreviated Scale of Intelligence II two test IQ (Wechsler, 1997); SSRT = stop signal reaction time; SST = stop-signal task; CF = cognitive flexibility; NB = n-back. Group mean is different from typical group at ***p < .001.

Participants were excluded from the study if they were reported to have head trauma, epilepsy, MRI scanner contraindications such as a non-removable metal implant, or vision that could not be corrected with MR-compatible glasses. Participants with an ADHD or comorbid diagnosis who were prescribed medication were instructed to follow their medication routine on the day of the scan. Thirteen individuals were on psychotropic medications during the scans; for details on medications, see Supplemental Table 1. Participants were included in the analysis if they had adequate data for at least one fMRI task, (see motion and performance cutoffs below in **Behavioral task analysis)**. The majority of the group (51 participants, >80%) contributed data for all three tasks, leaving a fairly consistent group of participants across the three tasks.

### 2.2 Symptom burden

Measures of symptom burden were collected from parents (parent child report: PCR) as part of the behavioral visit occurring 1–6 weeks before the MRI session. The Conners-3 (Conners, 2008) was used to measure ADHD symptom burden rating symptom burden on a scale from 0 to 3. Raw totals for predefined inattention and hyperactivity/impulsivity subscales were used to characterize ADHD.

### 2.3 fMRI tasks

MRI data were collected at the Biomedical Imaging Center at the University of Texas at Austin. All data were collected on a 3 T Siemens Skyra with a 32-channel head coil T1-weighted structural images were collected with an MPRAGE sequence (TR = 2530 ms, TE = 3.37 ms, FOV = 256, 1x1x1 mm voxels). T2-weighted structural images with a turbo spin echo sequence (TR = 3200 ms, TE = 412 ms, FOV = 250, 1x1x1 mm voxels) were collected. All functional scans used a multi-band echo-planar sequence (TR = 2000 ms, TE = 30 ms, flip angle = 60, multiband factor = 2, 48 axial slices, 2x2x2 mm voxels, base resolution = 128x128). Stimuli were presented via laptop with PsychoPy version 1.8 (Peirce, 2007) and projected at a resolution of 1920x1080 to a screen at the back of the MRI that participants viewed via a mirror attached to the head coil. Participants wore Optoacoustics headphones and microphone and provided within-task responses using a two-button response pad.

**Fig. 1:**
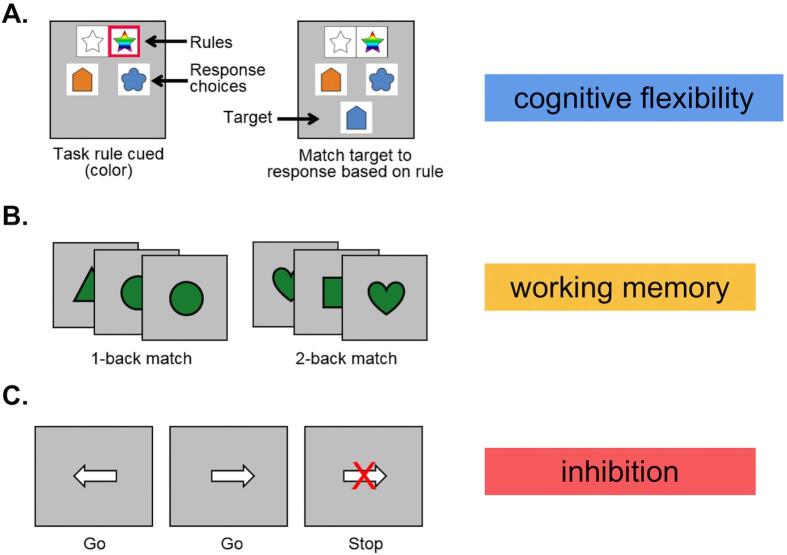
Example trials from the EF tasks. a. The cognitive flexibility task presented a red box around the relevant rule (shape or color) for 1500 ms, followed by a 500 ms delay, (0–2000 ms represented the cue period) and then the target appeared onscreen for 2000 ms (the target period). In this example trial, the relevant rule is “color”, and the correct answer for the target is the right response choice. b. The working memory task was an n-back task where participants responded only when the current stimulus matched either 1 before (left) or 2 before (right). c. The inhibition task was a stop-signal task that built up a “go” response to the direction of the arrows, and this response was interrupted in stop trials that were staircased in their onset (came faster or slower in the trial) depending on the participant's ability to stop at previous stop trials. See main text for details. All tasks are the same as (Engelhardt et al., 2019). (For interpretation of the references to color in this figure legend, the reader is referred to the web version of this article.)

Three tasks from distinct EF domains were presented in the following order (Fig. 1): cognitive flexibility (Bauer et al., 2017, Church et al., 2017, Engelhardt et al., 2019); working memory (Engelhardt et al., 2019); inhibition (Engelhardt et al., 2019, Roe et al., 2018). Task order was kept consistent across all participants and repeated once with a resting-state fMRI scan in between. Participants performed up to two runs of each task. The total session time was approximately 1.5 h, including resting state fMRI and diffusion tensor scans not discussed here.

*Cognitive flexibility task*: Participants performed an event-related design cued-rule matching task, where the relevant rule switched by trial (Church et al., 2017, Engelhardt et al., 2019) (Fig. 1). Each run consisted of 46 trials during which participants were cued to pay attention to either the shape or the color of a target stimulus that would appear on screen. The two ‘rules’ (shape or color) were on display throughout the duration of the trial. A red box indicating which rule to follow appeared around the trial-relevant rule for the first 1.5 s of the trial. On 80% of the trials, the target stimulus appeared 0.5 s (s) after the red box disappeared, and the target remained on the screen for 2 s. During this time participants used either the left or right button to indicate which of two choices above the target stimulus matched according to the current rule. All targets were incongruent, such that the two rules pointed at different answers, and thus attention to the relevant rule for that trial was critical. The response period was followed by a 1 s fixation cross. On 20% of trials, a target did not appear ('cue-only trials'), and a red fixation cross was displayed for 0.5 s, followed by a white fixation cross for 0.5 s. These ‘cue-only trials’ were used to separate the neural signal during the cue 'preparatory' period from the target 'task execution' period (Ollinger et al., 2001). Trials were followed by 0–8 s of fixation jitter. The total run time per task iteration was 5 min and 22 s. Sixty-two individuals out of the total 63 were included in the cognitive flexibility task analysis. One did not pass linear transformation quality assurance (QA) resulting from the bounding box not being applied correctly, and thus part of the image was clipped during collection.

*N-back working memory task*: Participants performed a block design n-back task to tax working memory (Engelhardt et al., 2019) (Fig. 1). 64 simple-colored shape stimuli were divided evenly into a 1-back and 2-back block per run. Blocks were preceded by a 4 s instruction screen indicating whether the participants should look for shapes that matched one shape before (1-back) or two shapes before (2-back). Stimuli were on screen for 1.5 s followed by a 1 s inter-stimulus interval. Participants were instructed to press a button when they saw a match for that block condition. A 20 s baseline rest block separated the 1-back and 2-back blocks as well as preceded and followed task blocks. Each block had a total of 7 matches, or 'hits' out of 32 stimuli per block (21.9% of trials). Working memory runs lasted 3 min and 32 s each. Sixty individuals out of the total 63 were included in the n-back task analysis. Three did not pass linear transformation QA resulting from the bounding box not being applied correctly, and thus part of the image was removed during collection.

*Stop-signal inhibition task*: A visual event-related stop-signal task was used to tax inhibition (Engelhardt et al., 2019, Rubia et al., 2003, Verbruggen and Logan, 2008) (Fig. 1). Each run consisted of 96 ‘go’ trials where participants pressed a button corresponding to the direction a horizontal arrow was pointing. The run also consisted of 32 ‘stop’ trials (25% of total trials) where a red X appeared after and on top of the arrow, cueing the participant to try and withhold a response and not press anything. For all trial types, arrows were displayed for 1 s, with a 1 s interval, followed by a jittered fixation of 0–6 s. ‘Stop’ trials were staircased in their timing; the first stop consisted of an arrow alone on the screen for 250 ms (the stop signal delay, SSD), before the red X appeared over the arrow and remained for the rest of the trial. If participants correctly stopped, the SSD on the next stop trial was increased by 50 ms (SSD = 300 ms). If the participant was unable to stop and made an incorrect button press, the SSD on the next stop trial decreased by 50 ms (SSD = 200 ms). This staircasing procedure continued throughout the duration of the task, with the goal of putting all participants at about 50% correct stop trials (6 min per run). Fifty-three individuals out of the total 63 were included in the stop-signal inhibition task analysis. Four did not complete the task, three were below task performance thresholds, and three did not pass linear registration QA resulting from the bounding box not being applied correctly, and thus part of the image was removed during collection.

### 2.4 Behavioral task analysis

Measures of task accuracy and response time (RT) were calculated for each task (Table 1). Overall accuracy and RT for correct trials was examined for the cognitive flexibility task. For the n-back task, the measures of interest were overall accuracy (hits and correct rejections) and RT for correct hits. These were calculated for each block (1-back and 2-back) separately, and the 2-back measures were used for this analysis. For the stop-signal task, measures of interest were accuracy on ‘go’ trials and stop signal reaction time (SSRT), which was calculated by taking the mean time between the presentation of the arrow and the appearance of an X (SSD) and subtracting it from the inter-quartile response time to a “go” trial. Since RT is known to have pronounced variability in ADHD (Kofler et al., 2013, Tamm et al., 2012), we also examined RT variability using the SD of RT for correct trials across the three tasks (SD of RT on go trials was used for the stop-signal task). Task behavioral measures were both correlated with ADHD symptom burden across the sample and tested for diagnosis group differences using the Welch’s *t*-test adjustment for unequal variances and unequal sample sizes in R version 3.2.1 (R Development Core Team, 2014).

To preserve broad individual differences in neural activity, performance cutoffs were more liberal than previously used with group models of these tasks (Engelhardt et al., 2019). Performance criteria was set to ensure participants understood and were engaged in the task and performing above chance on each task. Performance criteria was applied on a run-by-run basis. For the cognitive flexibility task, runs with <50% accuracy were excluded. Runs were excluded for the n-back task if 1-back hits <1 (i.e., must have at least 1 hit) or 1-back accuracy <50%. Stop-signal runs were excluded if ‘go’ trial accuracy was <50% or if mean SSRT was <50 ms. Thirteen total runs were excluded from these criteria, four from the cognitive flexibility task, one from the n-back task, and eight from the stop-signal task (four for go criteria, one for stop criteria, and three for both go and stop criteria). Task behavioral measures were tested for diagnostic group differences using the Welch’s *t*-test, and correlated with symptom burden using R version 3.2.1 (R Development Core Team, 2014).

### 2.5 MRI analysis

***Preprocessing:*** Imaging data were preprocessed using the FMRIB Software library (FSL) version 5.9 (www.fmrib.ox.ac.uk/fsl). T1 images were skull-stripped with non-brain matter removed using Freesurfer version 5.3.0 (Reuter et al., 2010). Registration of the high resolution structural to standard space was done with FMRIB’s Linear Image Registration Tool (FLIRT; (Jenkinson et al., 2002; Jenkinson and Smith, 2001). Images were spatially smoothed using a Gaussian kernel of FWHM 5 mm and the 4D dataset was grand-mean intensity normalized by a single multiplicative factor; high pass temporal filtering (Gaussian-weighted least-squares, straight line fitting, with sigma = 50 s).

***First level individual run modeling:*** Level 1 modeling was carried out in fMRI Expert Analysis Tool (FEAT). A double-gamma HRF time-series model was carried out using FILM with local autocorrelation correction (Woolrich et al., 2001). The highpass filter was set at 100 s for the switching and inhibition runs and to 200 s for the n-back runs, accounting for twice the duration of total stimulus presentation. First-level models included six motion regressors; temporal derivatives for each regressor (except for the n-back task, due to its block design); and nuisance regressors that censored individual volumes identified to have excessive motion, defined as framewise displacement greater than 0.9 mm (Siegel et al., 2014). See Supplemental Fig. 1 for % frames censored for each task. Task runs with <50% of frames remaining after motion censoring were not included in further analyses (N = 4 runs). All trials (correct and incorrect) from each task were combined in the analyses.

***EF contrasts of interest***: To test for the relation between symptom burden and EF neural activity, we chose five contrasts known to engage robust EF activity across the three tasks (Engelhardt et al., 2019). From the cognitive flexibility task, we chose a contrast combining the cue and target period (*whole trial vs. baseline*) as well as two contrasts testing the *cue (preparatory period) vs. baseline* and *target (task execution period) vs. baseline* separately*,* as the two have been shown to differ in control engagement (Church et al., 2017). For the working memory n-back task, we chose the *2-back block vs. baseline* contrast. For the inhibition stop-signal task, we chose the contrast *stop vs. baseline*. Second-level modeling across runs, averaged for each participant, was carried out by specifying a fixed effects structure within FMRIB Local Analysis of Mixed Effects (FLAME) (Beckmann et al., 2003). Higher-level group analyses for each task were carried out in FLAME. Statistical maps were thresholded with a cluster threshold of *Z* > 3.1, and whole-brain multiple comparisons were corrected using a cluster-level probability of *p* < .05 generated from Gaussian random field theory. These cluster-based thresholds were based on current best practices (Eklund et al., 2016, Woo et al., 2014).

### 2.6 Common EF activity across tasks

To examine EF activity common across the three tasks, we first replicated a previous cross-task overlap analysis using these same tasks, but modeled across error and correct trials, instead of just correct trials (Engelhardt et al., 2019). Our replication is done in a nearly non-overlapping group (one individual took part in both studies). Regions that showed common engagement across all three tasks in the current sample were termed the ‘core EF’ regions. These core EF regions were verified by comparing them to the location of 11 ROIs that showed consistent activity across the same three tasks in (Engelhardt et al., 2019); those 11 ROIs were used to test for BOLD activity relation to symptom burden. See Supplemental Methods section: Core EF regions of interest (ROI) analysis. For ROIs and coordinates, see Table 2.

**Table 2:** Core (three-task overlap) EF ROIs from Engelhardt et al. (2019) and overlapping three-task regions from the current study.

Engelhardt 2019 ROI	MNI coordinates			Current study nearest overlap peaks	MNI coordinates			Distance (in mm)	# Voxels
	X	Y	Z		X	Y	Z		
dorsal anterior cingulate	0	11	48	dorsal anterior cingulate	2	10	52	4.58	1085
left anterior insula	−31	20	3	left anterior insula	−30	20	6	3.16	261
right anterior insula	35	20	3	right anterior insula	34	20	4	1.41	395
right dorsal lateral PFC	37	33	28	right dorsal lateral PFC	42	30	28	5.83	65
right middle frontal gyrus	44	6	32	right middle frontal gyrus	40	4	42	10.95	805
right frontal eye field	26	−1	49	right middle frontal gyrus	40	4	42	16.43	805
left frontal eye field	−25	−5	51	left frontal eye field	−26	−4	52	1.73	85
left inferior parietal	−45	−39	43	left superior parietal	−32	−48	44	15.84	782
left superior parietal	−30	−49	45	left superior parietal	−32	−48	44	2.45	686
right inferior parietal	49	−41	47	right inferior parietal	56	−46	36	13.96	51
right superior parietal	33	−48	46	right superior parietal	34	−52	42	5.74	1331
–	–	–	–	right middle temporal gyrus	60	−31	−6	–	21
–	–	–	–	right thalamus	10	−12	10	–	131
–	–	–	–	right caudate	16	−2	18	–	22

Peak labels for the current study were taken from the Harvard-Oxford Atlas; PFC = prefrontal cortex. Engelhardt ROIs were also used for applied regional analyses for this study. Distance was calculated between Engelhardt 2019 sample ROIs and nearest overlap peak from the current study.

### 2.7 Whole-brain ADHD symptom burden analyses

To test for associations between individual differences in ADHD symptom burden and whole-brain activity for each EF task, mean-centered parent report of hyperactivity/impulsivity and inattention scores for each participant were added as a third-level correlate using FLAME stage 1. A *Z* > 3.1 threshold was used to define contiguous clusters with a cluster probability of *p* < .05 (Eklund et al., 2016). Gaussian random field theory was used for whole-brain multiple comparison corrections (Worsley, 2001). This was done separately for each of the three tasks. Separate statistical maps were generated for positive and negative correlations. Mean-centered age was added to all models to control for any age-related effects. All regions are reported in MNI coordinates and identified using the Harvard-Oxford Atlas in the FMRIB FSL-view software and Neurosynth (Yarkoni et al., 2011). Data were projected onto inflated brains maps for visualization purposes using Caret software (Van Essen, 2012).

### 2.8 Diagnostic group comparison models

In addition to our whole sample correlational models, we also took a more traditional approach of looking at ADHD effects in the brain by creating groups based on the presence or absence of ADHD diagnosis. Third-level models testing for group differences in mean activity between participants with an ADHD diagnosis and typically developing participants were carried out using FLAME stage 1. Mean-centered age was added to all models to control for any age-related effects. Group-level *Z* statistic images from each of the EF tasks were thresholded to correct for multiple comparisons (*Z* > 3.1, *p* < .05).

### 2.9 Restricted subgroup analysis

To examine potential effects of comorbid diagnoses and medication use, all of the same whole-brain symptom burden correlation models described above were run with all typically developing participants and a restricted subset of individuals who had an ADHD diagnosis but no comorbid diagnoses and were also not on any psychotropic medication at the time of scan. See Supplemental Methods & Materials and Results sections: Restricted comorbidity and medication subgroup analysis.

## 3 Results

### 3.1 Symptom burden

**Fig. 2:**
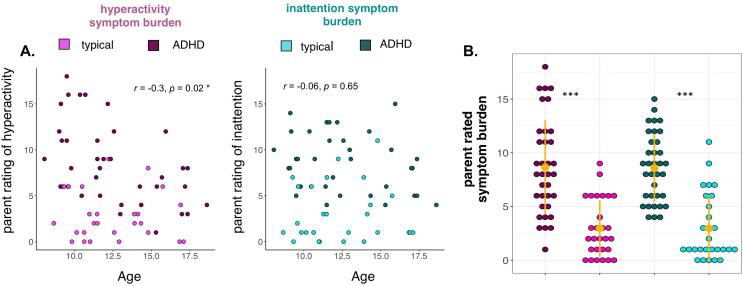
Parent ratings of ADHD symptom burden from the Conners-3 a. parent ratings of symptom burden plotted with age b. mean parent rated symptom burden. Error bars reflect standard deviation from the mean. * *p* < .05, *** *p* < .001. N = 63.

Parent ratings of ADHD symptom burden were higher for individuals with an ADHD diagnosis; this was true for both inattention (*t* = 7.12, *p* < .001) and hyperactivity (*t* = 6.33, *p* < .001) symptom ratings, though there was notable overlap (Fig. 2). Age was correlated with parent ratings of hyperactivity (*p* = .02), but not of inattention (*p* = .65). Parent ratings of inattention and hyperactivity symptom burden were correlated (*r* = 0.69, *p* < .001). There were no gender differences in parent reports of symptom burden (all *p*’s > 0.1).

### 3.2 Task data

*Cognitive flexibility*: There was a negative correlation between hyperactivity and accuracy on the task (*r* = −0.35, *p* < .01). There was no relation between inattention and task accuracy or between either measure of symptom burden and RT or RT variability (all *p*’s > 0.1). There were no differences in any performance measures between diagnosis groups (all *p*’s > 0.1, Table 1).

*Working memory*: There was no correlation between 2-back accuracy, hit RT, or hit RT variability and either measure of symptom burden. There were no diagnosis group differences in task performance between diagnosis groups (all *p*’s > 0.05, Table 1).

*Inhibition*: There was no correlation between SSRT, go RT variability, or go accuracy and either measures of symptom burden. There was no difference between diagnostic groups in task performance (all *p’*s > 0.1, Table 1).

### 3.3 Common EF activity across task

**Fig. 3:**
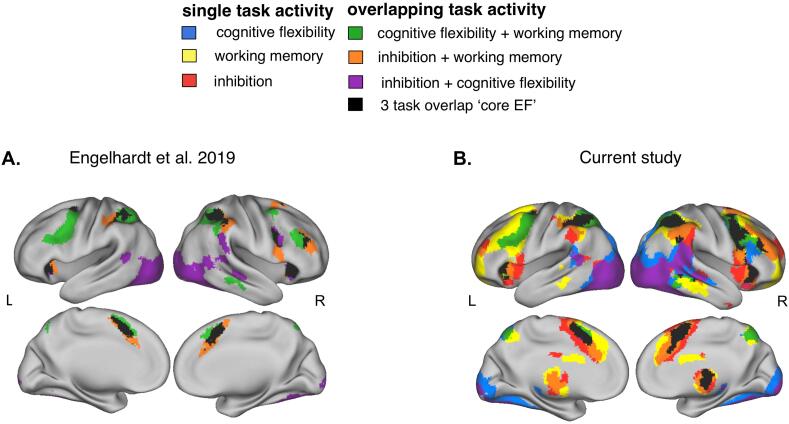
Overlapping regions of EF engagement across three tasks. For the three task Core EF overlap activity replication analysis; task-positive maps were binarized and overlaid to identify regions of activity common/overlapping across tasks. a. map of two- and three-task overlap activity from Engelhardt et al. 2019 (N = 117); b. full map of task overlap activity from the current study (N = 63); black represents three-task overlap activity used to generate overlap peaks; samples in each map >98% unique (one individual’s data is present in both maps).

Consistent with Engelhardt et al., 2019, we found a set of ‘core EF regions’ active across the three tasks in our sample (Flexibility: *cue vs. baseline*, Working Memory: *2back vs. baseline*, and Inhibition: *stop vs. baseline*; Fig. 3). These regions spanned dorsal attention, fronto-parietal, and cingulo-opercular putative control networks (Petersen and Posner, 2012, Power et al., 2011). All regions of the overlapping activity from the same contrast selection in Engelhardt et al., 2019 were less than 20 mm away from the centers of regions of overlapping activity in the current study (Table 2). See Supplemental Methods section: Common EF activity across tasks. Three unique spots of three-task overlap were found in our study: the right thalamus, the right caudate, and right middle temporal gyrus. Maps for each of the individual task contrasts of interest can be found in Supplemental Fig. 3.

### 3.4 ADHD symptom burden and EF task brain activity correlations

**Fig. 4:**
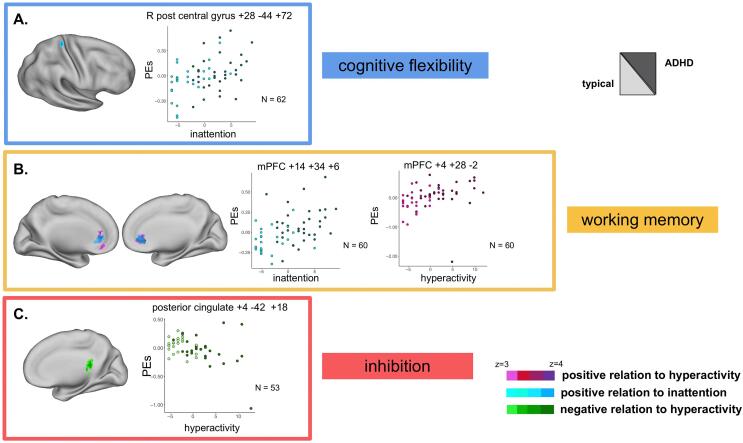
Parent-rated ADHD symptom burden correlated with neural activity across three EF tasks. Whole brain images and parameter estimates (PE) of brain activity plotted with measures of symptom burden from the whole-brain correlational models a. correlation between inattention symptom burden and activity during the cognitive flexibility task, *whole trial vs. baseline* contrast. b. correlation between hyperactivity or inattention symptom burdens and activity during the working memory task, *2-back vs. baseline* contrast. c. correlation between hyperactivity symptom burden and activity during the inhibition task, *stop vs. baseline and go vs. baseline* contrast. Mean centered parent symptom ratings and mean centered age were included in the models as covariates of no interest. All brain activity maps were cluster corrected for multiple comparisons at *Z* > 3.1 *p* < .05. Scatter plots merely depict whole brain correlations; no additional statistical tests were run on these data. mPFC = medial prefrontal cortex.

*Cognitive flexibility*: When testing the whole trial contrast (*whole trial vs. baseline*), controlling for age, there was a positive relation between inattention symptom burden and brain activity in right post-central gyrus and superior parietal lobe (Fig. 4a). See Table 3 for cluster size and locations. There was no relation to hyperactivity symptom burden. There was no relation between either inattention or hyperactivity symptom burden and neural activity during the cue period (*cue vs. baseline*) or target period (*target vs. baseline)* alone.

**Table 3:** Peak coordinates and cluster size from symptom burden correlation and group differences models.

Task	Symptom Burden	Correlation	Brain area	Peak Coordinates			Voxels
				x	y	z	
CF	inattention	positive	right post central gyrus	+28	−44	+74	104
	ADHD < no DX group difference		right occipital pole	+22	−98	−10	77
WM	hyperactivity	positive	ventral medial pre-frontal cortex (DMN)	+4	+28	−2	415
		positive	right orbitofrontal cortex	+28	+34	−2	102
	inattention	positive	medial prefrontal cortex (DMN)	+14	+34	+6	421
	ADHD > no DX group difference		medial prefrontal cortex (DMN)	+6	+26	−2	455
			medial anterior prefrontal cortex	−18	+50	+28	104
Inhibition	hyperactivity	negative	posterior cingulate (DMN)	+4	−42	+18	228

CF = cognitive flexibility (*cue vs. baseline*); WM = working memory (*2back vs. baseline*); cluster corrected for multiple comparisons; DMN = regions with negative activity, belonging to the default mode network; *Z* > 3.1 *p* < .05

*Working memory*: During the n-back task (*2back vs. baseline*), controlling for age, there was a positive relation between inattention and activity in the medial prefrontal cortex, and a positive relation between hyperactivity and activity in the medial prefrontal cortex, ventral medial prefrontal cortex, and right orbitofrontal cortex (Fig. 4b). Clusters for the hyperactivity and inattention models overlapped (42% of voxels overlapping relative to total active voxels). See Table 3 for cluster size and locations.

*Inhibition*: During the stop-signal task during the stop trials (*stop vs. baseline*), controlling for age, there was a negative correlation between hyperactivity and brain activity in the posterior cingulate gyrus (Fig. 4c). Mean activity in this region was negative, such that higher symptom burden meant greater deactivation of the region, in the opposite direction of what was seen in the working memory task. There were no significant results related to inattention symptoms.

*ROI analysis:* There were a few moderate correlations between activity in core EF regions and hyperactivity symptom burden during the cognitive flexibility and working memory tasks, after controlling for age (Supplemental Results section: Core EF regions of interest (ROI) ADHD symptom analysis). These relations were not consistently seen across EF domains and did not survive correction for multiple comparisons.

### 3.5 Diagnostic group comparisons

**Fig. 5:**
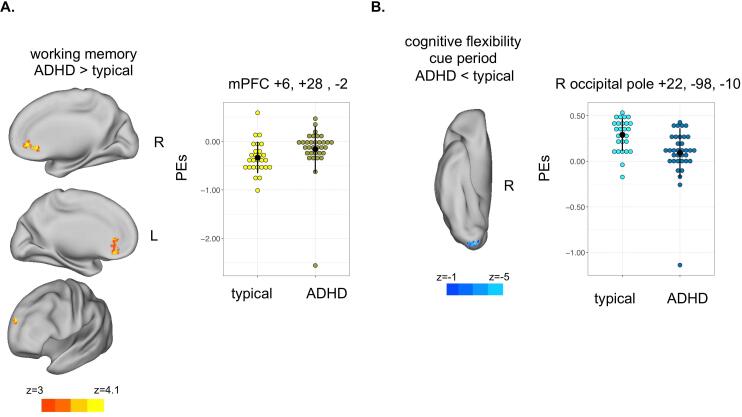
Group differences between individuals with an ADHD diagnosis (*n* = 35) and no diagnoses (*n* = 28). a. Whole brain group difference and parameter estimates (PEs) during a working memory task; b. Whole brain group difference and PEs during the cue period of the cognitive flexibility task; Hot colors represent ADHD > typical, cool colors represent ADHD < typical. All brain activity maps were cluster corrected for multiple comparisons at *Z* > 3.1 *p* < .05 with mean centered age included in the models. Error bars reflect standard deviation from the mean. Plots merely depict whole brain results; no additional statistical tests were run on these data. mPFC = medial prefrontal cortex.

During the cognitive flexibility task, individuals without an ADHD diagnosis had more activity in the right occipital pole, after controlling for age (Table 3, Fig. 5). During the working memory task, individuals with an ADHD diagnosis had less suppression of activity within the bilateral dorsal medial pre-frontal cortex (less negative activity) than did typically developing individuals (Table 3, Fig. 5), after controlling for age. These working memory task results were consistent to what was seen using continuous symptom burden ratings across the whole sample. No significant differences in brain activity were observed between diagnostic groups during the inhibition task.

## 4 Discussion

This work is uniquely positioned to examine the intersection of ADHD symptom burden, multiple EF domains, and brain engagement. By using within-sample correlation methods, we were able to capitalize on individual differences in symptom burden across a varied group and to examine the impact of distinct types of ADHD symptoms. We addressed two main questions: First, in a large pediatric group with varied ADHD symptom burden, do two facets of ADHD differentially relate to neural activity in three EF tasks? We found some overlap between inattention and hyperactivity measures during our working memory task, but otherwise found distinct symptom results, with a notable lack of consistent pattern of ADHD-brain activity correlations across the three EF tasks. Second, if we do see relations between symptom burden and brain activity, are a core set of putative control regions (i.e., those active across all EF domains) carrying those effects? We found brain activity primarily in the default mode network (DMN) as well as somatosensory and visual regions to vary with symptom burden and did not observe such patterns in control regions.

### 4.1 Relations between brain activity and ADHD vary across EF domains and types of symptom burden

We present experimental evidence within the same pediatric sample that the relations between ADHD symptom burden and brain activity are EF task specific. We found unique associations between brain activity and symptom burden in tasks related to inhibition, cognitive flexibility, and working memory, with no overlap. Our results suggest that ADHD symptom burden does relate to EF brain function, but that distinct EF domains are impacted in different ways. This is supported by a meta-analysis that found different associations between ADHD and brain activity during inhibition versus attention tasks (Rubia et al., 2012) and aggregated ADHD studies on neurosynth.org. This distinction is important when considering the heterogenous phenotypic expressions of ADHD in control-demanding settings such as the classroom. Understanding EF heterogeneity in ADHD could be key to designing more targeted supportive and remedial programs addressing and individual’s specific deficits (Chacko et al., 2014).

The current work sheds some light on the variability in brain regions that are reported across the ADHD neuroimaging literature. This heterogeneity in the literature is exemplified by use of ‘Neurosynth’ (Yarkoni et al., 2011), a meta analytic tool which provides statistical maps of voxels related to a given term from a large corpus of neuroimaging papers. A current search of the term ‘ADHD’ reveals 144 studies with 3888 reported activations but a practically empty main statistical map, meaning that there is very little consistency in the active voxels reported. Our work suggests that some of that lack of consistency may be due to studies using single task collections of different types of tasks such as inhibition or working memory, and thus tapping into different effects of ADHD. Even in the same group of individuals, we find non-overlapping effects related to both categorical (diagnosis) and dimensional (symptom burden) aspects of ADHD across different tasks tapping EF. This lack of overlap is likely reflected in the literature more broadly as even meta analyses find non-overlapping effects across groups of studies using different types of tasks (Rubia et al., 2012). Future studies of the brain and ADHD should carefully consider the choice of task and cognitive domain they are measuring in their group. We join others (Karalunas & Nigg, 2020) in recommending collection of multiple tasks spanning several cognitive domains in order to capture heterogeneity in brain systems varying with ADHD. We also encourage the use of both dimensional and categorical measures of ADHD in the same group as our work joins previous work (e.g., Chabernaud et al., 2011) in highlighting unique effects found when testing categorical *vs*. dimensional measures.

### 4.2 ADHD symptom burden and brain activity relationships are seen outside of core EF regions

Considering the reported relation between EF performance and ADHD in children (Kofler et al., 2018, Nigg et al., 2005), we expected that activation in ‘core EF’ regions, which are uniformly engaged across control-demanding tasks by middle childhood, would vary in accordance with ADHD symptom burden. While we did replicate consistent engagement of these regions across EF tasks in our current unique sample, we did not find that activation in these regions varied with ADHD symptom burden; there was no consistency to ADHD correlations across tasks. Because engagement of these putative EF brain networks appears in place by mid-childhood, it may be less vulnerable to ADHD symptom burden than expected. An important next avenue towards brain profiles in ADHD would be to test the coordination between the overall putative EF system and the large-scale systems (i.e., sensorimotor, default mode network) that did vary with ADHD symptom burden in this sample.

### 4.3 Task preparation and execution vary with ADHD

In our cognitive flexibility task, we saw increased activity in somatosensory regions related to inattention symptom burden. Previous work showing atypical activity in somatosensory systems suggests an over-reliance on these systems when task preparation isn’t properly initiated (Fassbender et al., 2009), symptomatic of more reactive control in children with higher ADHD symptoms. This is further supported in our work by isolating the cue period of the flexibility task, during which we found diagnostic group differences in occipital cortex such that individuals with ADHD have lower levels of activity in the region. This result in the cue period suggests children with ADHD have different preparatory strategies than children without ADHD. Previous work using a similar paradigm has found adults engaged occipital regions more than children during the cue period (Church et al., 2017); children also had other evidence of less preparation during the cue period in that study. The occipital difference in this study could reflect compounded immature preparation in those with ADHD, leading to the need for the more reactive control to execute the task, as we see in somatosensory and anterior parietal cortex across the trial. Coordination between preparatory and execution control systems required to carry out a task could thus be especially vulnerable to attention difficulties. Tasks that can isolate preparation and execution periods are a valuable and underutilized paradigm for studying coordination between these systems.

### 4.4 Default mode network regions vary with ADHD symptom burden across multiple EF domains

The default mode network (DMN) results seen in both our inhibition and working memory tasks, in posterior cingulate cortex and medial pre-frontal cortex respectively, are consistent with a large literature indicating attention problems and ADHD impact DMN function (Castellanos and Proal, 2012, Cortese et al., 2012, Fair et al., 2010). During the working memory task, we find that individuals with lower symptom burden deactivate the medial prefrontal cortex regions more than with individuals with higher symptom burden, while we see the opposite in the inhibition task. These findings are in line with the ‘default mode interference hypothesis,’ wherein coherence of the default mode network, as well as its connections to other brain systems, is disrupted in ADHD, and may affect shifts between rest to attention states (Raichle and Snyder, 2007, Sonuga-Barke and Castellanos, 2007). This hypothesis has been supported in previous work showing alterations both in activity (Fassbender et al., 2009, Whitfield-Gabrieli and Ford, 2012) and connectivity (Castellanos and Proal, 2012, Chabernaud et al., 2011, Fair et al., 2010, Kelly et al., 2007, Konrad et al., 2006, Whitfield-Gabrieli and Ford, 2012) of the DMN in populations with ADHD. Interestingly, we did not see consistent direction or location of effects across EF tasks, even within the default mode network. This could be due to different subsystems of the DMN being recruited for each EF task or varying differently with ADHD (Gordon et al., 2020). The lack of consistency in the extant ADHD neuroimaging literature is likely due to multiple sources, but our work reveals that correlations between ADHD symptoms and brain activity even within one brain system appear to be task specific.

### 4.5 Hyperactivity, inattention, and diagnostic group difference effects overlap during working memory

This work highlights the diversity in relationships between dimensions of ADHD behavior and brain activity across domains of EF, highlighting the need for careful task selection and generalization of findings. While we see remarkably different results across the three domains in the location and nature of attention relations, our working memory domain results were consistent across types of symptom burden (hyperactivity and inattention) and were robust across subgroup analyses. Specific working memory deficits in ADHD have received notable attention in recent years (Fosco et al., 2020, Kasper et al., 2012, Kofler et al., 2010). In behavioral work measuring across domains of EF in ADHD, working memory deficits have been reported to be higher than deficits in other domains (Karalunas et al., 2017, Kofler et al., 2018). Working memory deficits in ADHD have been linked to both hyperactivity (Rapport et al., 2009) and inattention (Karalunas et al., 2017, Kofler et al., 2010) symptoms. Given this multidimensional overlapping relationship between ADHD symptom burden and working memory, the executive processes underlying working memory might be particularly important avenues for studying ADHD in childhood and adolescence (Fosco et al., 2020).

### 4.6 Limitations

The current work has several notable limitations. In order to keep protocols consistent, all tasks were presented in the same order for each participant. This led to slightly less collection of the second run of the last task (stop-signal inhibition task). Varying task order in future studies could mitigate this issue and result in more even coverage across tasks. Additionally, task performance thresholds were kept liberal to preserve broad individual differences. Lowering performance thresholds introduces more possibilities of including noisier data from when participants not engaged in the task. However, we found no relationship between inattention symptom burden and task performance, indicating we don’t have a systemic issue of including individuals with high inattentive burden by lowering performance thresholds. Given that individual differences in attention is a core construct of interest in this study, we decided lowering thresholds was important for retaining variability in participant sampling. Future studies of ADHD dimensions in larger samples could compare results from varying performance criteria and inclusiveness. Due to MRI bounding box limitations in this study, we had inadequate coverage of the cerebellum and were unable to include it in the current study. The cerebellum has been shown to vary anatomically and functionally with ADHD diagnosis and symptomology (Fair et al., 2012, Krain and Castellanos, 2006, Rubia et al., 2009b). Future neurobiological studies of EF and ADHD should test for consistent aberrant activity or connectivity patterns of the cerebellum.

We did not impose medication restrictions on our participants, thereby creating a heterogenous sample. We made this decision to avoid transient withdrawal effects and to increase the comfort and inclusion of participating families. To address this possible confound, we tested for effects in a smaller unmedicated, ADHD-only subsample, but at the expense of substantial loss of power. We believe our community-recruited sample reflects the high degree of ADHD medication use and comorbid diagnoses with ADHD in ‘real-world’ samples, and thus reflects many experiences with ADHD. We cannot definitively reject the possibility that our lack of consistent across-task results was driven by the effects of medication or comorbid diagnoses; similarly, these factors may have masked additional meaningful results from the current study. Future work with larger samples such as the National Institute of Health's Adolescent Brain Cognitive Development study drawn from cross-site collections (Casey et al., 2018), may shed additional insight on this question. However, our tests of three different EF tasks within the same set of participants uniquely positioned us to cleanly address the question of cross-task consistencies. Despite keeping all study parameters across tasks consistent, cross-task overlap with ADHD hyperactivity and inattention symptoms was not observed.

## 5 Conclusions

Across tasks from three EF domains, we did not find a coherent pattern of associations between brain engagement and ADHD symptom burden. We also did not see consistent cross-task ADHD symptom relations in putative core EF regions that are active across the three tasks. We found task-specific correlations between symptom burden and brain activity in somatosensory, visual, and DMN regions. This work provides evidence of the neural heterogeneity of EF function related to attention difficulties within a single sample. Our results strongly support testing multiple EF domains and using both categorical and dimensional measures of ADHD in future brain and behavioral studies.

## Financial disclosures

The current study was supported by Jessica A. Church’s Brain & Behavior Research Foundation NARSAD Young Investigator Award, and University of Texas start-up funds. The content is solely the responsibility of the authors and does not necessarily reflect the views of the funders.

## CRediT authorship contribution statement

**Tehila Nugiel:** Conceptualization, Methodology, Formal analysis, Investigation, Data curation, Writing - original draft. **Mary Abbe Roe:** Data curation, Methodology, Writing - review & editing. **Laura E. Engelhardt:** Methodology, Writing - review & editing. **Mackenzie E. Mitchell:** Data curation, Project administration, Writing - review & editing. **Annie Zheng:** Data curation, Project administration, Writing - review & editing. **Jessica A. Church:** Supervision, Funding acquisition, Writing - review & editing.

## Declaration of Competing Interest

The authors declare that they have no known competing financial interests or personal relationships that could have appeared to influence the work reported in this paper.
