# Supplementary material for: Pediatric ADHD symptom burden relates to distinct neural activity across executive function domains
Source: Neuroimage Clin. 2020 Aug 25;28:102394. doi: 10.1016/j.nicl.2020.102394 (PMC7511724; doi:10.1016/j.nicl.2020.102394)
Supplement: Supplementary data 1 [file mmc1.pdf]

Nugiel, T., Roe, M. A., Engelhardt, L. E., Mitchell, M. E., Zheng, A., & Church, J. A. (2020). Pediatric ADHD symptom burden relates to distinct neural activity across executive function domains. *NeuroImage: Clinical*, 102394.

Supplemental Materials

Supplemental Methods and Materials

Medication	N
stimulant ADHD	22
nonstimulant ADHD	9
other psychotropic medication	7
more than one medication	9
no medication	9

Supplemental Table 1. *Psychotropic medication usage in the ADHD diagnosed (N=35) sample.* Medications are listed for any individual who reported currently taking psychotropic medication (N=26). Of note, some medicated participants reported not taking medication on the day of scanning (N=13 of 35 reported use of psychotropic medication on the day of the scan session). Nine children diagnosed with ADHD were completely medication-free. Nine children were on more than one medication (reported within stimulant and nonstimulant totals above; five were on a combination of ADHD medications and four were on a combination of ADHD and other psychotropic medications). Non-diagnosed participants (N=28) were not on any psychotropic medications.
Stimulant ADHD medications included Adderall, Concerta, Daytrana, Focalin, and Ritalin.
Non-stimulant ADHD medications included Vayrin, Intuniv, Strattera, and Clonidine.
Other psychotropic medication included selective serotonin reuptake inhibitors, serotonin norepinephrine reuptake inhibitors, norepinephrine reuptake inhibitors, and antipsychotics

Additional Diagnosis	N
Anxiety	2
Dyslexia	4
Autism Spectrum Disorder	2
Tourette Syndrome	2
Bipolar Disorder	1
Obsessive Compulsive Disorder	1

Supplemental Table 2. *Comorbid disorders with ADHD in the ADHD-diagnosed sample (N =35).* Ten children had an additional diagnosis beyond ADHD. Two children had more than one comorbid diagnosis.

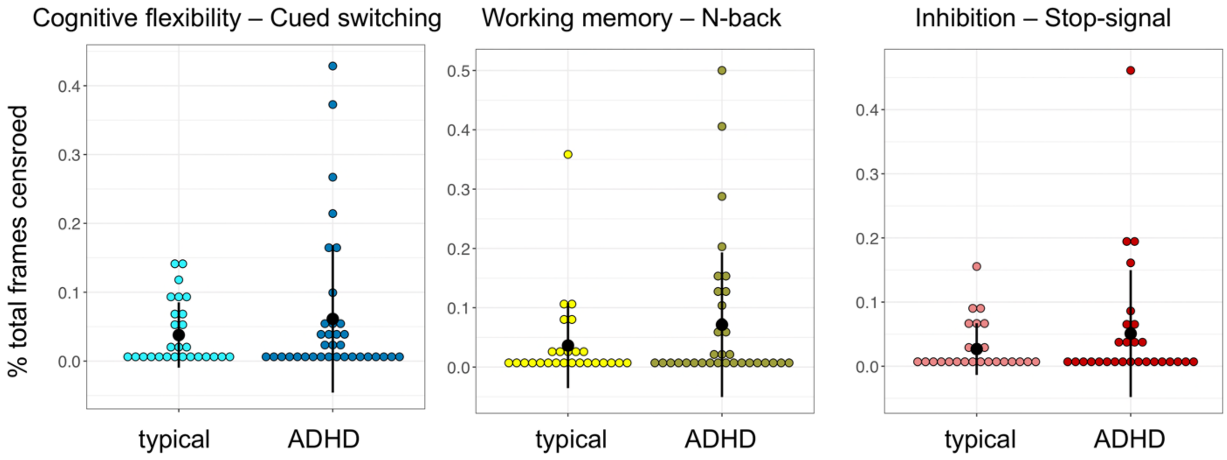

Supplemental Figure 1. *Frames scrubbed from each task.* Percent of total frames collected censored at FD > 0.9 mm plotted for each individual by diagnostic group. Errors bars represent standard deviation from the mean. Plots reflect all collected data for the analyzed sample (N=63) before any runs were dropped (for high overall motion, poor task performance, or preprocessing problems - see main text for run exclusions). There were no significant group differences in % censored frames or post-scrubbed motion (*p's*> .05).

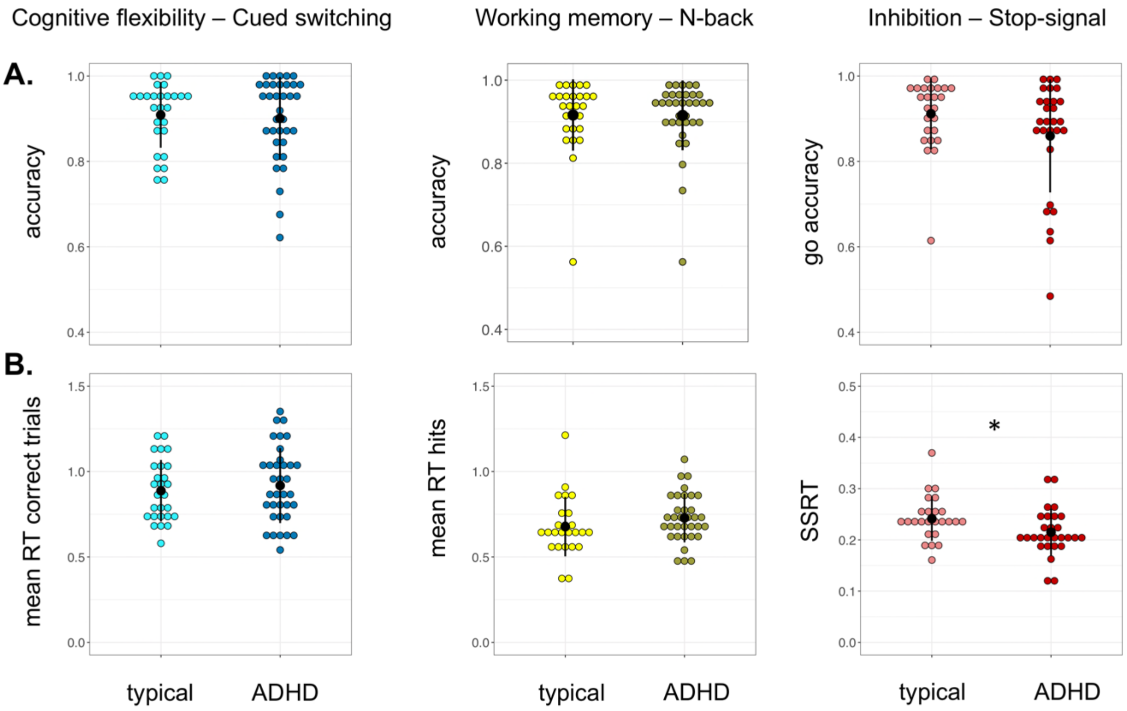

Supplemental Figure 2. *Behavioral performance for each task*. a. Accuracy on the three tasks plotted for each individual by group; b. Response time for the three tasks plotted for each individual by group; Errors bars represent standard deviation from the mean; SSRT group means are different at *$p < .05$ (*);* Plots reflect all collected data for the full sample (N=63) before specific runs were dropped due to task performance criteria (see main text).

Common EF activity across tasks

Methods for creating maps of common EF activity across tasks were closely modeled after previous work with the same tasks (Engelhardt et al., 2019). The samples (n=117, Engelhardt et al., and n =63, current sample) overlapped by 1 person. A difference from the analysis of Engelhardt and colleagues, which examined overlap across correct trials only, was that this analysis examined activity across all trials. One contrast from each task (*cue vs. baseline* from the cognitive flexibility task, *2-back block vs. baseline* from the N-back task and *stop vs. baseline* from the SST*)* was used to test for common EF activity across domains (following Engelhardt et al., 2019). Contrasts were thresholded at $Z > 3.1$ and $p < .05$ (Supplemental Figure 3).

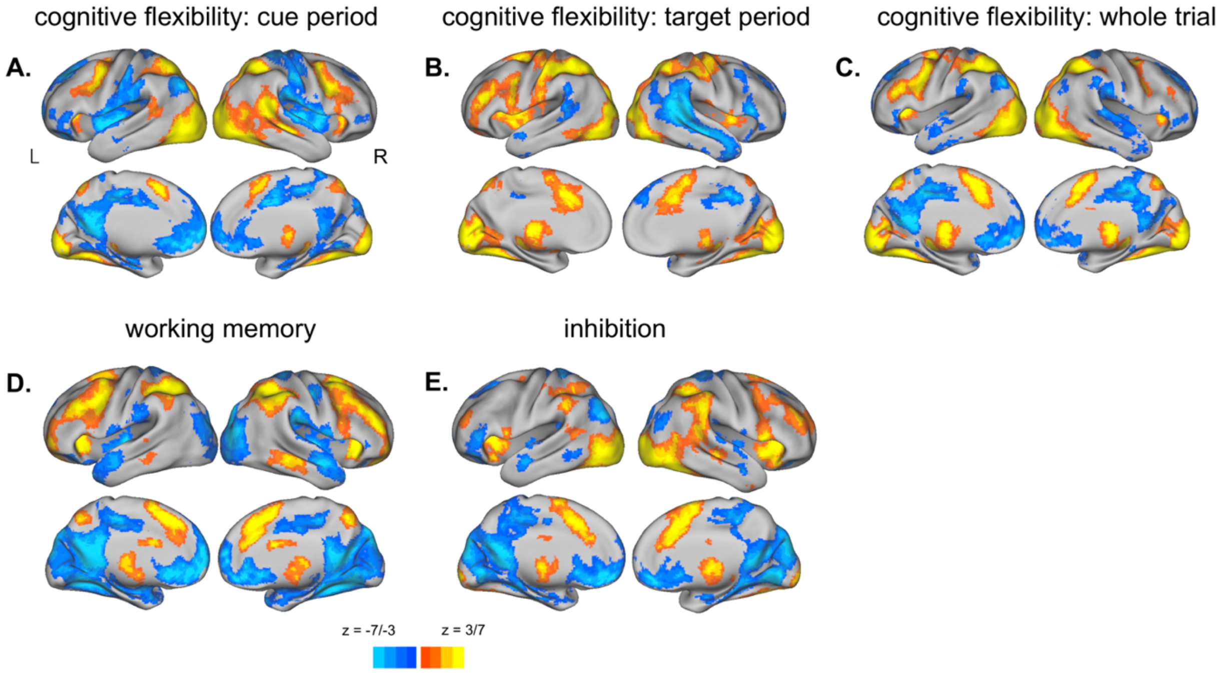

Supplemental Figure 3. *Main effect contrasts of interest from three EF tasks across the whole sample (N= 62 for the cognitive flexibility task, N = 60 for the working memory task, N = 53 for the inhibition task).* a. *cue vs. baseline* from the cognitive flexibility task b. *target vs. baseline* from the cognitive flexibility task c. *whole trial vs. baseline* from the cognitive flexibility task d. *2-back vs. baseline* from the working memory task e. *stop vs. baseline* from the inhibition task

Maps of positive tasks activity (activity greater than baseline) were then binarized. The three binarized maps from each task were overlaid to create one map of overlapping and unique task activity. To measure the distances between the ROIs from the previous study (Engelhardt et al., 2019) and our clusters of overlapping activity across the three tasks FSL *Cluster* tool was used to identify coordinates for the center of each cluster within the summed task-overlap mask. The distance between ROIs from the previous study and the center of clusters from the current study was computed as:

$$\textit{distance (mm)} = \sqrt{(x_A - x_B)^2 + (y_A - y_B)^2 + (z_A - z_B)^2}$$

where x, y, and z correspond to the MNI coordinates for the current study's centers of activity (A) and previously seen pediatric ROIs (Engelhardt et al., 2019) (B).

Core EF regions of interest (ROI) analysis

To test for relations between ADHD symptom burden and brain activity in core EF regions, the 11 ROIs from the previous study (Engelhardt et al., 2019) that captured the core EF activity in an independent pediatric sample were used in an applied ROI analysis (see Study 1, Table 2 for ROIs and coordinates). ROI analyses were carried out using FSL and R. The 11 sets of coordinates were used to create 5 mm radius ROIs using the T1 MNI152 2mm mask in FSL. To test for relations between brain activity in these ROIs and symptom burden, ROIs were applied to the BOLD activity of each of the three EF tasks. Neural activity parameter estimates for each contrast were calculated for each ROI, for each individual. One sample two-tailed t-tests were used to assess whether ROIs had a mean activity across individuals that was significantly different from 0. Semipartial correlations controlling for age were estimated between parameter estimates from the ROIs and raw inattention and hyperactivity PCR scores.

Restricted comorbidity and medication subgroup analysis

To examine potential influence of comorbid diagnoses and medication use, all of the same whole-brain symptom burden correlation models described in Materials & Methods were run with all typically developing participants and a restricted subset of individuals who had an ADHD diagnosis but no comorbid diagnoses and were psychotropic medication-free at time of scan (N=45 across the whole group, N= 44 for the cognitive flexibility task, N = 42 for the working memory task, N =39 for the inhibition task). Mean centered age was included as a covariate of no interest in all models.

Supplemental Results

Core EF regions of interest (ROI) ADHD symptom analysis

First, we tested that there was significant engagement of the applied regions in the contrasts. Seven of the 11 applied ROIs had mean activity > 0 during the target period of the cognitive flexibility task (all but the right middle frontal gyrus, right anterior insula, right dorsal lateral prefrontal cortex, and right inferior parietal ROIs). Ten of the 11 had mean activity > 0 during the cue period of the cognitive flexibility task (all but the right inferior parietal lobe ROI). All 11 of ROIs had mean activity > 0 during the working memory task. Ten of the 11 had mean activity > 0 during the inhibition task (all but the right frontal eye field ROI). All p's < .05 corrected for multiple comparisons.

Before correction for multiple comparisons, during the target period of the cognitive flexibility task, the frontal eye field ($r = .28$, $p = .03$) showed a positive relation to hyperactivity symptom burden. There were no relations to inattention symptom burden. There were no

relations between either inattention or hyperactivity ADHD symptom burden and the ROIs during the cue period or whole trial of the cognitive flexibility task (all *p's* > .1). During the working memory task hyperactivity was negatively related to the left inferior parietal lobe (r = -.26, p = .05). All reported values are uncorrected for multiple comparisons, and these effects did not survive FDR multiple comparison correction. There were no relations to inattention symptoms (lowest p = .08 uncorrected). No relation was seen for any ROIs during the inhibition task contrast and either measure of ADHD symptom burden (lowest p = .13 uncorrected).

Restricted comorbidity and medication subgroup analysis

Restricted comorbidity and medication use subgroup whole brain analyses

Fewer effects were observed in the restricted (no comorbidities, no medication) subgroup analysis, likely due to the smaller size of the groups (Supplemental Figure 4; Supplemental Table 3). Importantly, removing medication use and comorbidities did not make any task-ADHD relations more similar to each other across different tasks. During the cognitive flexibility task (*whole trial vs. baseline*) there was a negative relationship between inattention and neural activity in a region spanning the right orbital frontal cortex and right anterior insula, as well as a region of the dorsal-lateral prefrontal cortex During the working memory task, there was a positive correlation between hyperactivity and neural activity in a similar region of medial prefrontal cortex to the models with the full group and a novel region in the lateral left anterior prefrontal cortex. Also, in the working memory task, there was a positive correlation between inattention symptoms and neural activity in the right frontal pole. There was no relation between the BOLD contrast of the inhibition task and either ADHD symptom profile in this subgroup.

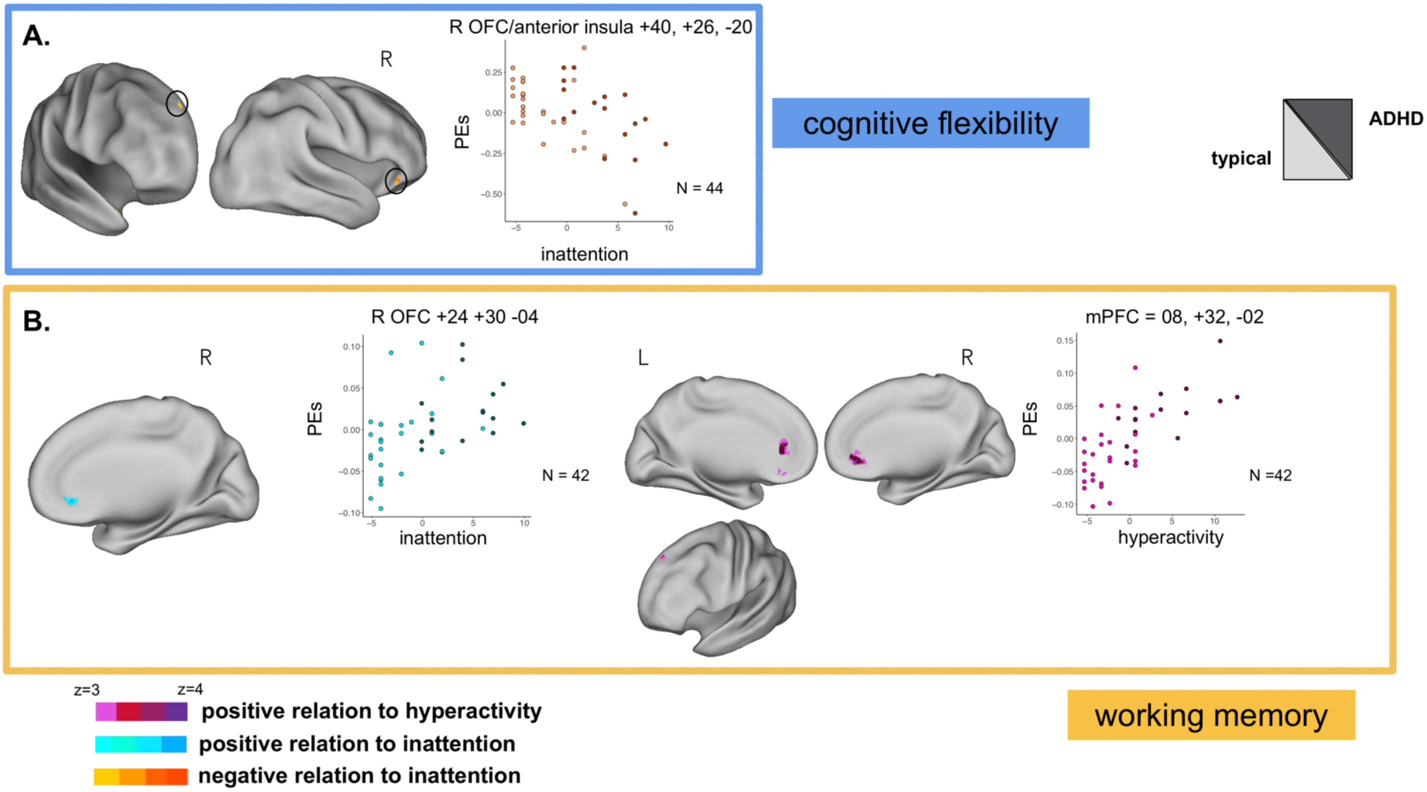

Supplemental Figure 4. *Whole brain restricted comorbidity and medication subgroup analysis: Parent-rated inattention and hyperactivity symptom burdens correlated with neural activity across the EF tasks.* Whole brain images and parameter estimates (PE) of brain activity plotted with mean-centered measures of symptom burden, controlling for age, from the whole-brain correlational models. a. Correlation between inattention symptom burden and the cognitive flexibility task *(whole trial vs. baseline*) resulted in a right OFC region; b. Correlation between hyperactivity and inattention symptom burden during the working memory task *(2back vs, baseline)* resulted in mPFC regions. No significant results were found in the inhibition task. Scatterplots merely depict whole brain correlations; no additional statistical tests were run on these data. mPFC = medial prefrontal cortex; OFC = orbital frontal cortex.

Task	Symptom Burden	Correlation	Brain area	Peak Coordinates			No. of voxels
				x	y	z	
CF	inattention	negative	right ventral frontal cortex/inferior insula	+40	+26	-20	101
			right anterior prefrontal cortex	+20	+38	+40	98
WM	hyperactivity	positive	medial prefrontal cortex	+8	+32	-2	339
			left anterior prefrontal cortex	-22	+40	+50	83
			right orbital frontal cortex	+28	+34	-2	83
	inattention	positive	right ventral medial prefrontal cortex/orbital frontal cortex	+24	+30	-4	127

Supplemental Table 3. *Peak coordinates and cluster size from symptom burden correlations in restricted subgroup (N=44 for the cognitive flexibility task, N = 42 for the working memory task) whole brain analysis*. CF = cognitive flexibility (*whole trial vs. baseline*); WM = working memory (*2back vs. baseline*); age was included in the covariate models; maps were cluster corrected for multiple comparisons z > 3.1 p <.05; coordinates are reported in standard MNI space

Restricted comorbidity and medication use subgroup ROI analyses

The same significant main effect engagement of applied ROIs was seen in the restricted subgroup as in the larger group above. Before correction for multiple comparisons, three ROIs showed correlations to hyperactivity symptom burden across the task contrasts. During the target period of the cognitive flexibility task, the left frontal eye field (r = .37, p = .01) and the left insula (r = .39, p = .009) were related to hyperactivity, while during the working memory task the right middle frontal gyrus was negatively related to inattention (r = - .35, p = .02). There was no relation between the ROIs during any task and inattention symptom burden. Just as in the larger group, the symptom profile effects were not consistent across EF domains and did not survive correction for multiple comparisons.
